# Building back better children's surgical services toward universal health coverage: Perspectives from Bangladesh and Zimbabwe

**DOI:** 10.3389/fpubh.2023.1073319

**Published:** 2023-01-25

**Authors:** Dennis Mazingi, Tanvir Kabir Chowdhury, Tasmiah Tahera Aziz, Nowrin Tamanna, Kokila Lakhoo, Tahmina Banu, Saqif Mustafa

**Affiliations:** ^1^Nuffield Department of Surgical Sciences, University of Oxford, Oxford, United Kingdom; ^2^Nuffield Department of Surgical Sciences, Medical Sciences Division, University of Oxford, Oxford, United Kingdom; ^3^Department of Surgery, College of Health Sciences, University of Zimbabwe, Harare, Zimbabwe; ^4^Department of Paediatric Surgery, Chittagong Medical College and Hospital (CMCH), Chattogram, Bangladesh; ^5^Chittagong Research Institute for Children Surgery (CRICS), Chattogram, Bangladesh; ^6^Department of Surgery, University of the Witwatersrand, Johannesburg, South Africa; ^7^Muhimbili University of Health and Allied Sciences, Dar es Salaam, Tanzania; ^8^Global Health Policy Unit, The University of Edinburgh, Edinburgh, United Kingdom

**Keywords:** universal health coverage (UHC), health systems recovery, children's surgery, access to health services, global surgery, COVID-19, Zimbabwe, Bangladesh

## Abstract

**Introduction:**

This article is part of the Research Topic ‘Health Systems Recovery in the Context of COVID-19 and Protracted Conflict’. Children's surgical services are crucial, yet underappreciated, for children's health and must be sufficiently addressed to make and sustain progress toward universal health coverage (UHC). Despite their considerable burden and socioeconomic cost, surgical diseases have been relatively neglected in favor of communicable diseases living up to their inauspicious moniker: ‘the neglected stepchild of global health'. This article aims to raise awareness around children's surgical diseases and offers perspectives from two prototypical LMICs on strengthening surgical services in the context of health systems recovery following the COVID-19 experience to make and sustain progress toward UHC.

**Approach:**

We used a focused literature review supplemented by the perspectives of local experts and the 6-components framework for surgical systems planning to present two case studies of Bangladesh and Zimbabwe. The lived experiences of the authors are used to describe the impact of COVID-19 on respective surgical systems and offer perspectives on building back the health system and recovering essential health services for sustainability and resilience.

**Findings:**

We found that limited high-level policy and planning instruments, an overburdened and under-resourced health and allied workforce, underdeveloped surgical infrastructure (from key utilities to essential medical products), lack of locally generated research, and the specter of prohibitively high out-of-pocket costs for children's surgery are common challenges in both countries that have been exacerbated by the COVID-19 pandemic.

**Discussion:**

Continued chronic underinvestment and inattention to children's surgical diseases coupled with the devastating effect of the COVID-19 pandemic threaten progress toward key global health objectives. Urgent attention and investment in the context of health systems recovery is needed from policy to practice levels to improve infrastructure; attract, retain and train the surgical and allied health workforce; and improve service delivery access with equity considerations to meet the 2030 Lancet Commission goals, and make and sustain progress toward UHC and the SDGs.

## Introduction

Universal health coverage (UHC) means that all people including children have access to quality health services without financial hardship (1). Surgery and surgical health services have been recognized as an essential part of UHC by the World Health Organization (WHO) and World Bank (2). Despite this, the importance of surgical care has been underappreciated for several decades due to a preferential focus on infectious diseases in the public health discourse. The burden of surgical disease in children is considerable—one third of childhood deaths in the world are attributable to surgical conditions—and with expected surge in child population in Africa, this figure can be expected to increase (3). It has been estimated that about 85% of children in low- and middle-income countries (LMICs) have surgically correctable conditions by the age of 15 years (4). However, two thirds of the world's children, mostly in LMICs, do not have access to surgical care (5). Every year over 77.2 million disability-adjusted life-years (DALYs) could be averted by basic, life-saving surgical care (6). Furthermore, through early prevention and corrective interventions, costly secondary and tertiary interventions at later stages in the life course can be averted for improved health and wellbeing, enhanced socio-economic prospects, and reduced healthcare costs. Bangladesh and Zimbabwe are two LMICs that have made impressive improvements in maternal and child health, however, there remains a large unmet need for surgical services with children in both countries facing similar challenges globally. This article examines the state of children's surgical conditions in both countries, outlines the gaps that still exist and describes the devastation wrought by the COVID-19 pandemic in this key area of global health that has been neglected for decades. The article offers perspectives from two LMICs on strengthening surgical services in the context of health systems recovery following the COVID-19 experience to make and sustain progress toward UHC. It draws heavily on The Lancet Commission on Global Surgery report—a landmark publication that outlined the scale of the previously underappreciated problem and laid out global aspirations for scaling up access to surgical care to underserved regions by 2030 (7). It is a roadmap for global surgical efforts that described core indicators for monitoring of universal access to safe, affordable surgical and anesthesia care.

## Approach

We conducted focused literature reviews adapting an approach which has been utilized previously in considering global surgery in a health systems context (8). This involved searching PubMed and Google Scholar with review of the first five pages of sources for each of the 6 components in the framework for surgical systems planning to categorize the effects of COVID-19 on surgical systems in the target countries (Table 1) (7). The findings were supplemented by the perspectives of local experts to identify key vulnerabilities in surgical systems of each country (Bangladesh and Zimbabwe). Key vulnerabilities were identified, discussed and presented for each country. The countries represent two diverse LMIC regions (Sub-Saharan Africa and South-East Asia, respectively) and have evolving demographics reflecting the broader global south, i.e., growing young populations and likely increased prevalence of children's surgical conditions in the twenty-first century.

**Table 1:** The 6-component framework for surgical systems planning (7).

**Component**	**Indicators**
1. Infrastructure	• Proportion of the population with 2-h access to a first-level facility • WHO Hospital Assessment Tool (a structured appraisal of equipment, electricity, water and sundries) • Proportion of hospitals fulfilling the safe surgery criteria • Blood bank donation rate and distribution
2. Workforce	• Density and distribution of specialist surgical, anesthetic and obstetric (SAO) providers • Number of SAO graduates and retirees • Proportion of surgical workforce training programmes accredited • The presence of task sharing or nursing accredited programmes and number of providers • The presence of attraction and retention strategies • Density and distribution of nurses, and ancillary staff including operational managers, biomedical engineers, and radiology, pathology, and laboratory technicians
3. Service delivery	• Proportion of surgical facilities offering the Bellwether procedures • Number of surgical procedures done per year • Peri-operative morbidity and mortality • Availability of system-wide communication
4. Financing	• Surgical expenditure as a proportion of gross domestic product • Surgical expenditure as a proportion of total national healthcare budget • Out-of-pocket expenditures on surgery • Catastrophic and impoverishing expenditures on surgery
5. Information management	• The presence of data systems that promote monitoring and accountability related to surgical and anesthesia care • Proportion of hospital facilities with high-speed internet connections
6. Governance	• Governmental and non-governmental actors that influence SOA health delivery structures • The manner in which these key actors relate and engage with another to influence health delivery • Formulation of policies, regulations, and national budgets

Zimbabwe and Bangladesh were chosen because they are the home countries of the authors who have on-the-ground experience as well as understanding of sociocultural norms and the local health system. In the context of extremely scarce research on children's surgical care in both countries, an approach of using evidence augmented by the insights of experts and vice versa is a pragmatic solution in developing initial recommendations for policy, practice and future research.

## Findings

### Bangladesh

Bangladesh has around 64 million children that make up 38.64% of the population (9). Despite this, pediatric surgical services are distributed unequally. The majority of services are only available in urban areas, especially in the large tertiary hospitals (10). Otherwise, pediatric surgical services are provided for by general surgeons in peripheral district hospitals. Bangladesh has made good progress toward UHC with its UHC index improving from 38 in 2010 to 49 out of 100 in 2020.

#### Infrastructure

**Figure 1:**
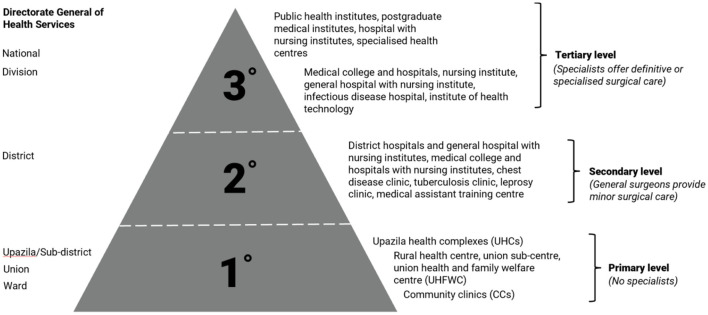
Hierarchy of Bangladeshi healthcare facilities under the directorate general of health services (11).

Similar to many LMICs the country has a hierarchical health system with primary health up to specialist-level hospitals. Peripheral care at subdistrict level is provided at Upazila Health Complexes (UpHCs) (Figure 1). The healthcare infrastructure under the Directorate-General of Health Services (DGHS) includes six tiers: national, divisional, district, upazila (subdistrict), union, and ward facilities that map onto the traditional three-tier system of care as follows: the upazila, union, and ward offer primary care; the district tier offers secondary care; and the divisional and national tiers offer tertiary care (Figure 1) (11). In 2013 there were 436 Upazila Health Complexes, 53 district hospitals, nine general hospitals and 12 specialized hospitals. Only 44% of UpHCs had a functioning anesthetic machine while Oxygen and a functioning anesthesia machine were unavailable in 14% of district and general hospitals (11). There is a shortage of neonatal and pediatric surgical intensive care facilities, total parenteral nutritional support availability, and oxygen supply in peripheral health facilities (expert observations).

#### Workforce

A considerable number of minor surgical procedures are performed by village doctors, paramedics and unqualified persons in peripheral health institutions (12–14). Not much national level data on the total number of pediatric surgical providers is available. In 2015 Bangladesh had 161 pediatric surgeons (0.3 pediatric surgeons per 100,000 population under the age of 15 years) with a deficit of 375 needed (15). In 2022, there are ~205 pediatric surgeons in the country (0.43 pediatric surgeons per 100,000 population under the age of 15 years) working in 22 public and 10 non-government healthcare facilities (personal communication).

It has been observed that more junior cadres (resident doctors) played a key part in service provision during the pandemic echoing the importance of human resources as a building block of successful health systems (16). Pre-pandemic shortage of pediatric and neonatal anesthetists, pediatric surgical nursing staff and pediatric pathologists is also the norm (expert observation).

#### Service delivery

Elective surgeries were pre-emptively canceled indefinitely across many countries in an effort to free up surge capacity and manpower as well as to reduce the risk of nosocomial COVID-19 infection in patients (8). In a nationwide survey of pediatric surgeons 75% of respondents revealed that they had experienced a drastic decline in surgical volumes of up to 70% while another study demonstrated similarly drastic declines in admissions (59%), outpatient attendance (72%) and elective surgery (83%) (17). Surgical volumes are a key indicator for success in the Lancet Commission report and during the pandemic reflected altered decision making by surgeons as well as reduced access.

#### Financing

The health financing system in Bangladesh heavily relies on out-of-pocket expenditure. About 72.9% of the health expenditure comes out-of-pocket, which is highest in the South Asia Region (18). A study from Bangladesh found that out-of-pocket cost for Inguinal hernia surgery is minimal [USD 5.3] when done as outreach surgical service (19). Reducing the financial burden of surgical care remains a major priority for LMICs.

The cost per disability adjusted life-year for a variety of surgical conditions in both countries such as hernias, appendicitis and abscess surgery compares favorably with other well known public health interventions (20). Children's cancer care is also very cost effective, contradicting the common fallacy that this kind of care does not provide value for money (21).

#### Information management

There is a wide disparity in availability of information technology equipment between public and private facilities. According to the Bangladesh health facility survey 95% of private facilities and 82% of district and UpHCs have a functional land-line or mobile phone while only 3% of union-level facilities and CC's do (11). Similarly, only 22% of union-level facilities have a functioning computer with Internet access while 91% of district and UpHCs and 75% of private hospitals do have access (11).

#### Governance

There is no national surgical obstetric and anesthesia plan (NSOAP) in Bangladesh, which is a first step to prioritizing surgery at a governance level. The Global Initiative for Children's Surgery (GICS) published a seminal document “Optimal resources for Children's Surgical Care” contributed by both high-income country (HIC) and LMIC providers where guidelines for different Levels of Care, Supplies, Equipment, Infrastructure and Research can be found (22).

### Zimbabwe

Zimbabwe, similar to Bangladesh as well as many developing countries, is characterized by a young, growing, mostly rural (67%) population (23). People in the more remote rural areas have relatively less access to health facilities than their urban counterparts and have to travel longer distances to access care. There is a significant burden of both infectious and noncommunicable diseases as expected for an industrializing country in epidemiologic transition (23). The pace of UHC has slowed in recent years with the country's UHC index having remained around 55 percent since 2015 despite initial success.

There is scanty data on the burden of surgical disease in the country; however existing data reveals that the burden of injury, childhood cancer and congenital malformations is considerable. Injury is a major cause of death in children and adolescents. It is the second highest cause of death among children 5–14 years of age in Harare and has risen steadily in the rankings in the past decade (24). Road traffic accidents (RTAs) are the most common cause of injury in the country and are steadily rising (25). Zimbabwe is among countries with the highest age-standardized DALY rates (26) with an incidence of childhood cancer of 120 per million in 2013. Malignancies are the 5th highest cause of death in Harare in the 5–14-year age group (26). In Zimbabwe, as in many African countries, late presentation and constrained access to cancer care services are common and predictably lead to worse outcomes (26).

#### Infrastructure

##### General infrastructure in Zimbabwe

**Figure 2:**
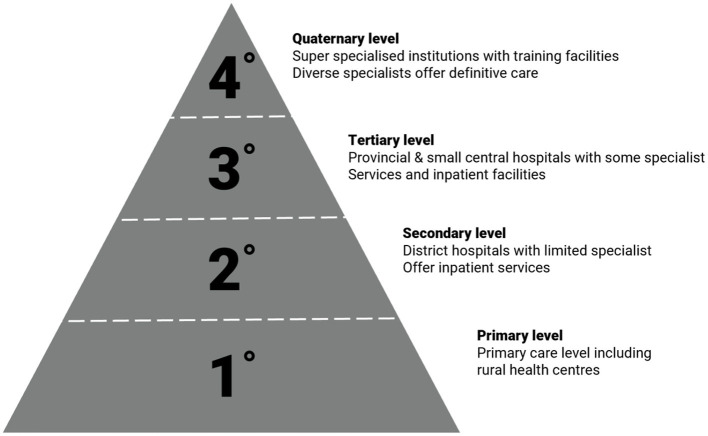
Hierarchy of Zimbabwean healthcare facilities under the Ministry of Health (27).

Zimbabwe's health system has four hierarchical tiers of care that include primary, secondary, tertiary and quaternary levels as shown in Figure 2. Surgery for children of varying complexity is performed at all levels of this system ranging from basic procedures to complex, specialist surgery at the quaternary level. In 2015 there were 1,848 healthcare facilities in the country including 6 central hospitals, 8 provincial hospitals and 44 district hospitals (27).

##### Surgical infrastructure in Zimbabwe

Only 44% of all healthcare facilities in Zimbabwe offer basic surgical services that run the gamut from wound cleaning to incision and drainage and closed repair of a fracture. These are primarily offered at the higher tiers of care. Wide disparities exist in public vs private and urban compared to rural facilities. A much lower percentage of the country's hospitals have the capability to provide comprehensive surgical care. In a survey from 2018, 100% of hospitals had some form of oxygen and only 15% of both district and mission hospitals lacked a functioning anesthetic machine (28).

There are currently only two dedicated children's hospitals in the country, with child-only operating theaters. These are located in Harare and Bulawayo, two major cities. The concentration of children's services in one dedicated service facilitates synergies between specialities and increasingly specialized care, however, the separation of services has been cited as an impediment to timely care.

#### Workforce

Zimbabwe has faced a critical healthcare workforce shortage in recent years exacerbated by economic decline and brain drain. However, the expansion of the College of surgeons of east, central and southern Africa (COSECSA) training program has increased the surgical workforce in recent years (expert observation). Zimbabwe currently has 5 consultant pediatric surgeons; however, adult surgeons also perform surgery in children (expert observation). This is an increase from two in 2015 and shows that modest progress has been made. The pediatric surgical workforce density is still short of what it should be. The cadres who perform surgery for children in the country include consultant pediatric surgeons and adult surgeons, medical officers, and surgeons-in-training. The surgical workforce is being continually augmented by the training of non-specialist doctors in basic surgical care as part of the Zimbabwe essential surgical training initiative. As of 2018, 102 non-surgeons were trained under this program (unpublished data). During the pandemic healthcare worker industrial actions further constrained surgical care during the pandemic, in addition to COVID-specific factors (expert observation).

#### Service delivery

Service delivery for children's surgery experienced a precipitous drop during COVID-19 (29). In a recently published study, the weekly median surgical volume in Zimbabwe dropped from 37.5 to 13 procedures per week (29). The proportion of electives of total procedures dropped from 8.2 to 0% during the first 6 months of the pandemic (29). The low preCOVID-19 rate of elective procedures was notable because of the healthcare industrial actions that took place in the year and months preceding the pandemic. This will also have implications for the ability of the surgical system to rebound after the pandemic (30).

#### Financing

In Zimbabwe as in Bangladesh, asymmetries in protection by government subsidies, a high unemployment rate and high rates of poverty leave many households potentially vulnerable to catastrophic health expenditures (31). Out-of-pocket expenditure for healthcare in Zimbabwe is high, reaching 24% of health expenditures in 2015 (31). In 2018 total out-of-pocket expenditure (OOPE) for healthcare in Zimbabwe was estimated at 343.7 million USD, translating to 24.90 USD per capita per year (31). The per capita OOPE was around 10% of average personal 2018 monthly income but taking into account the significant wealth inequality, could amount to more than 100% of income for those people who attend public healthcare facilities (expert observation). Granular data for OOPE for surgical conditions in Zimbabwe does not currently exist but injury and neoplasms together account for 10.84% of total OOPE (31). Mechanisms of financial support to reduce out-of-pocket expenditure for healthcare that have been used in the country include vouchers for blood use in maternity care, donor-support for radiological investigations and medication in childhood cancer and waiving of user fees for under-5 children (expert observation).

#### Information management

Zimbabwe has a relatively robust health information system that collects routine health facility data from all levels of the health system to the Ministry of Health (32). However, there is no trauma registry nor is there a congenital diseases registry in the country that would contribute to high quality outcome and clinical data on these key pediatric surgical conditions. The country does have a long-standing cancer registry that includes pediatric tumors (33). Communication between rural and district surgeons and specialists in urban central regions increased during the pandemic as well as a surge in telemedicine utilization (expert observation). These changes were facilitated by updating of regulations for telemedicine during the pandemic (34) as well as a pragmatic response to changed referral patterns during the pandemic (8). Published data on facility communication infrastructure or internet is not available.

#### Governance

Zimbabwe has recently completed and released its national surgical obstetric and anesthesia plan and is notably one of the few countries that have incorporated children in their NSOAS from inception (35, 36). This is a crucial first step to prioritizing surgery and bringing surgical disease to the fore in the country and demonstrates the government's commitment to the cause of global surgery. The development process was delayed by the COVID-19 pandemic which prevented teams from meeting and competed for policy and decision-making capacity (expert observation).

## Discussion

A key step toward improving children's surgical services in the LMIC context is through institutionalized planning and policy instruments such as NSOAPs. Bangladesh does not currently have an NSOAP. A dedicated plan for surgical and obstetric care, that prioritizes children can put this neglected area on the radar of key decision makers at local, national and global levels. Furthermore, it can be utilized to bring the surgical community together in the country to advocate and leverage national ansd international funding, resources and in-kind support. Simply having a dedicated NSOAP, however, will not be enough to bring much needed attention to the children's surgery agenda. There will need to be specific considerations for common children's surgical conditions in line with national contexts in each NSOAP. Furthermore, the plans will need to be adequately funded with credible implementation plans in a reasonable time and provision made for monitoring and evaluation. Importantly, the NSOAP should not exist or be implemented in isolation and should link to multi-year national health policies and plans, and their planning cycles, such as the national health sector strategic plan and essential package of health services.

Impacts on the SAO workforce were particularly influential in curtailing surgical care during the pandemic. This in addition to the pre-emptive cancellation of children's surgery was devastating to the 15-year campaign to expand access to surgery globally described by the Lancet Commission on Global Surgery report. Cancellations of elective surgery were common around the world but were not supported by evidence. The risk of infection in children remained low in Bangladesh (37) Cancellations in children in particular are controversial because children's beds did not provide useful surge capacity for COVID-19 in the target countries (29). And children have comparatively low perioperative mortality after COVID-19 infection (38). The excess mortality caused by untreated surgical disease may therefore still exceed the potential risk of COVID-19 related nosocomial infection or perioperative COVID-related death.

Governments should consider protection of SAO workers as the most important resource during and outside of pandemics (22). The promotion of their mental health and safety and retention is of utmost importance. The pandemic revealed preexisting vulnerabilities and stressed SAO workers to the limit. A more sustainable healthcare workforce will contribute to resilience for future pandemics. The Lancet Commission report made no specific mention of children's surgery, an important oversight. There is an urgent need to develop indicators for children's surgical care in addition to those for adults as well as bellwether procedures for children.

A common theme in both countries is the opportunity borne out of necessity for increased utilization of information and communication technologies (ICT) to facilitate links between central hospitals and distant facilities in districts and rural areas. Use of consultation prior to surgery, planning of surgery and decision-making for transfer and patient flow are just some of the applications of ICT that can mitigate the negative effects of the pandemic and improve efficiency and effectiveness in health systems.

The paucity of research and granular data (incomplete, non-interoperable, inaccessible and unpredictable) at subnational, community and rural levels is a barrier to the development and implementation of an evidence-based National Surgical, Obstetric and Anesthesia Plan with necessary considerations and resources for children's surgery in Bangladesh and other LMICs. For implementation of the NSOAP and scale up of children's surgical services, there is a need to conduct population needs assessment and cost assessment. It is criticaI to understand the service needs according to target population criteria such as children's age, sex, socioeconomic and education group, religion, geographical location (e.g., urban, rural, or tribal) and health sector (e.g., public, private, not-for-profit, informal).

There exist noteworthy limitations in the deliberations and conclusions drawn in this perspective piece including the risk of bias (e.g., citation bias) using non-systematic methods There is currently a dearth of literature in global pediatric surgery especially relating to the impacts of the COVID-19 pandemic in these countries and the effects varied widely across many LMICs. This manuscript is aimed at emphasizing those unique impacts in the context of Zimbabwe and Bangladesh and may have relevance for other LMIC country contexts as they progress with health systems recovery and making and sustaining progress toward UHC.

## Conclusion

Bangladesh and Zimbabwe are examples of LMICs with a significant burden of pediatric surgical disease with major implications for broader individual and population health, psychosocial health and economic development. Current surgical services are unable to meet this demand with improvements needed in quality, access, equity, financial protection aligned with universal health coverage/sustainable development goals. The pandemic severely limited health system capacity for surgery and surgical systems resilience has been tested to the limit during this period. Urgent resuscitative attention and investment is needed from policy to practice levels to improve infrastructure, attract, retain and train the surgical and allied health workforce and improve service delivery access with equity considerations to meet the 2030 goals of the Lancet Commission report and make and sustain progress toward UHC.

## Data availability statement

The original contributions presented in the study are included in the article/supplementary material, further inquiries can be directed to the corresponding author.

## Author contributions

TC and DM contributed to conception, design of the study as well as literature review, and manuscript writing. KL and TB were involved in concept development, manuscript writing, and critical review. TTA, NT, and SM were involved in data acquisition and critical review. DM wrote the first draft of the manuscript. TTA, NT, SM, and TB wrote sections of the manuscript. All authors contributed to manuscript revision, read, and approved the submitted version.
